# Systematic Review and Meta-analysis: Changes of Gut Microbiota before and after Menopause

**DOI:** 10.1155/2022/3767373

**Published:** 2022-07-25

**Authors:** Meina Yang, Shu Wen, Jing Zhang, Jin Peng, Xiaoyang Shen, Liangzhi Xu

**Affiliations:** ^1^Department of Obstetrics and Gynecology, West China Second University Hospital, Sichuan University, Chengdu, China; ^2^Reproductive Endocrinology and Regulation Laboratory, West China Second University Hospital, Sichuan University, Chengdu, China; ^3^Key Laboratory of Birth Defects and Related Diseases of Women and Children (Sichuan University), Ministry of Education, Chengdu, China; ^4^The Joint Laboratory for Reproductive Medicine of Sichuan University-The Chinese University of Hong Kong, China

## Abstract

**Objective:**

To systematically evaluate the differences in intestinal flora before and after menopause. To provide a possible mechanism for perimenopausal syndrome and provide a basis for probiotics as adjuvant therapy.

**Methods:**

MEDLINE, EMBASE, Web of Science, Cochrane Central Register of Controlled Trials (CENTRAL), CNKI, Wanfang, and VIP databases were searched. The included studies were case-control studies.

**Results:**

Three case-control studies were included, with a total of 156 people. At the phylum level, there were no differences between premenopausal and postmenopausal women. At the genus level, the relative abundances of A. odoratum and B. cholerae were higher in postmenopausal women than in premenopausal women, with no differences among other genera. The Shannon diversity index increased after menopause, but no differences were found. Only one study found a positive association of estradiol with Gammaproteobacteria and Myxococcales and a negative association with Prevotellaceae.

**Conclusions:**

On the basis of previous studies, it was found that there was no significant difference at the phylum level between postmenopausal women and premenopausal women, but Odoribacter and Bilophila increased at the genus level in postmenopausal women. The class of Gammaproteobacteria may be positively correlated with estradiol. Limited by the number of included studies, more high-quality clinical studies are needed for validation.

## 1. Introduction

There are about 1.5 million women getting into menopause every year [1]. It has become an intriguing problem to pay attention to postmenopausal women and their socioeconomic benefits. The most obvious change in postmenopausal women is the decline of ovarian function, resulting in estrogen fluctuations. Studies have shown that intestinal flora may be related to estrogen: intestinal flora with estrogen metabolism-related gene (estrobolome) can pass through *β* glucosidase which “activates” the uncoupled bound form of estrogen, thereby increasing the free estrogen in the hepatointestinal circulation. These estrogens bind to receptors and play the role of stimulating follicular development and promoting bone density, cardiovascular protection, etc. [2]. At present, there are few articles on the relationship between estrogen and intestinal flora. Only the following three articles are human observation experiments: Shin et al. divided the subjects into the low, medium, and high groups according to the level of sex hormone. The results show that the level of estrogen is related to the diversity and composition of intestinal microorganisms; that is, in women aged 25-65, the level of serum estradiol is significantly negatively correlated to *Slackia* and *Butyricimonas* [3]. The paired study of pre- and postmenopausal women by Santos-Marcos et al. found that the level of serum estradiol was positively correlated with *Gammaproteobacteria* and an unknown genus of *Myxococcales* and negatively correlated with *Prevotellaceae* [4]; Zhu et al. found that the level of estradiol was weakly positively correlated with *Shewanella putrefaciens* and *Erwinia amylovora* [5]. It was shown that the estrogen level may be related to the intestinal flora, so the change of intestinal flora may be related to the change of menopause and even related to menopausal syndrome, such as osteoporosis, metabolic abnormalities, depression, Alzheimer's disease, and urogenital symptoms [6–8]. Based on this, it is necessary to study the differences in intestinal flora before and after menopause.

This article is aimed at investigating whether there are differences in the abundance of gut microbiota before and after menopause and whether sex hormones are related to gut microbiota. This meta-analysis is aimed at finding possible clues about the etiology and mechanism of menopause-related diseases and providing evidence for probiotics such as probiotics and prebiotics as adjuvant therapy for menopause.

## 2. Materials and Methods

### 2.1. Inclusion and Exclusion Criteria

We follow the protocol registered with PROSPERO (registration number: CRD42021251454). Studies that met the following inclusion criteria were included: (1) The study type was a case-control study. We defined premenopausal women as the control group and postmenopausal women as the case group. (2) Subjects were premenopausal and postmenopausal women. Menopause is defined as “excluding women who are pregnant, over 40 years old, and over 12 months after the last menstrual period”. (3) The results include the relative abundance and diversity index of each intestinal flora (Shannon index, Chao1 index). The Shannon index is the heterogeneity index of the community, *H* = ∑(PI) (ln PI), where PI represents the proportion of the *i*th species in the total number [9]; the Chao1 index is the index of species richness, Chao1 = *n* + (*N*1∗(*N*1 − 1))/(2∗(*N*2 + 1)), where *n* is total out, *N*1 is the number composed of one read, and *N*2 is the number of 1 read and the correlation coefficient with estradiol [10]. The exclusion criteria were as follows: (1) subjects had used antibiotics or probiotics within 3 months and (2) studies that met the criteria were included when the same studies were encountered.

### 2.2. Data Extraction

Two researchers independently assessed articles and collected information. Inconsistencies were judged by discussion with the assistance of a third author. Two researchers independently collected the following data: basic characteristics (author, year, and country), subject characteristics (age, number of people in each group), gut microbiota analysis method (sample type, storage conditions, DNA extraction method, sequence analytical techniques, and reference databases), and results. When the article did not provide data, we extracted data from the images via ImageJ and used a digitizer. We emailed the authors for more information.

### 2.3. Search Strategy

Databases include MEDLINE (via Ovid, 1974-2020), EMBASE (via Ovid, 1974-2020), Cochrane Central Register of Controlled Trials (CENTRAL) (via Ovid), Web of Science, CNKI, Wanfang, and VIP. The search period is 1974 to March 27, 2021, with no language limitation. Search terms include menopause, postmenopause, premenopause, castration, oophorectomy, salpingo-oophorectomy, gastrointestinal microbiome, gut microbiome, Firmicutes, Bacteroidetes, Proteobacteria, stink bacteria, Prevotella, Parabacteroidetes, Satrera, and Brautia.

### 2.4. Quality Assessment

Two researchers independently assessed the quality of the literature according to the Newcastle-Ottawa Scale (NOS) score for case-control studies, and agreement with the third author was required in case of discrepancies. In the study, a score of 0-3 was low quality, a score of 4-6 was medium quality, and a score of 7-9 was high quality.

### 2.5. Data Synthesis and Analysis

Meta-analysis was performed using Review Manager 5.3 software. Differences of Means (MDs) are used when continuous variables (flora abundance, Shannon index, and hormones) are involved. The inconsistency index (*I*^2^) was used to test for heterogeneity. When *I*^2^ < 50%, a fixed-effects model was used; otherwise, a random-effects model was used. Two-tailed values of *P* < 0.05 were defined as statistically significant. Data synthesis was performed with reference to the method of Kim et al. [11]. Sources of heterogeneity were sought through Stata 12.0 metaregression and sensitivity analysis. Publication bias was assessed by funnel plots using Review Manager 5.3.

## 3. Results

### 3.1. Study Selection

**Figure 1:**
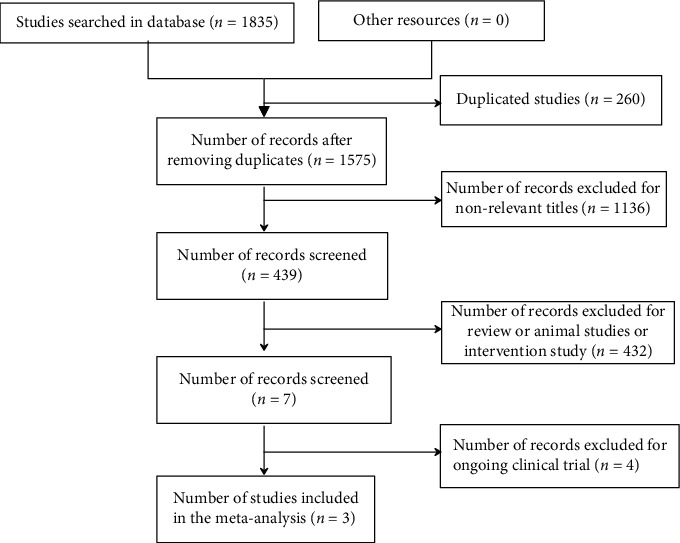
The flow chart.

A total of 1835 references were screened, and 260 duplicated studies were screened out by Endnote x8 software. After reading the title and abstract, we excluded 1136 irrelevant articles. After checking the full text according to the above inclusion and exclusion criteria, 3 studies were included [4, 5, 12]. The flow chart is shown in Figure 1.

### 3.2. Characteristics of the Studies

Three case-control studies were included, one of which was Caucasian (17 nonmenopausal, 20 postmenopausal) and two East Asians (25 and 24 premenopausal, 46 and 24 postmenopausal). A total of 156 women were included, including 66 premenopausal women and 90 postmenopausal women. Three studies reported on bacterial abundance, of which two studies reported *α* Shannon indices [5, 12] and one study reported the Chao1 index [5].

According to the NOS score, one article was rated as 7 points [4], which was considered a high-quality study. The scores of the other two articles were 4 and 6 [5, 12], respectively, which were considered to be of medium quality. The basic characteristics and quality of the included studies are shown in Table 1.

### 3.3. Sample Handling and Microbiological Evaluation Methods

Sample analysis and data processing for each study are shown in Table 2. All three articles used stool samples, and two of them mentioned the storage conditions (-80°C) of the samples. Two studies mentioned DNA extraction protocols and kits. Sequencing was provided in all 3 studies, two of which were metagenomic sequencing and one was V1-V2 region sequencing. Two studies provided reference databases, and two studies used multiple comparisons tests.

### 3.4. Changes in Intestinal Flora before and after Menopause

**Figure 2:**
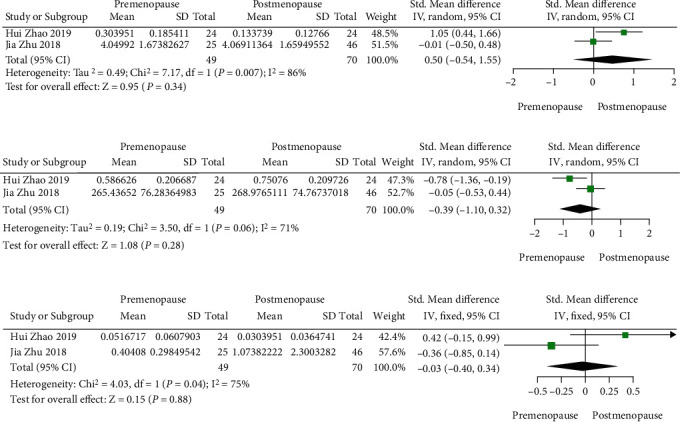
(a) *Firmicutes*; (b) *Bacteroides*; (c) *Proteobacteria.*

Two studies reported the changes in abundance at the phylum level, three studies reported the changes in abundance at the genus level, and one of them also reported the changes at the species level. There are no differences in *Firmicutes* (SMD = 0.50, 95%CI (−0.54, 1.55), *P* = 0.34), *Bacteroides* (SMD = −0.39, 95%CI (−1.10, 0.32), *P* = 0.28), and *Proteobacteria* (SMD = 0.03, 95%CI (−0.40, 0.34), *P* = 0.88) between pre- and postmenopausal women, while *Bacteroides* and *Proteobacteria* show a downward trend and *Firmicute*s presents an upward trend (Figures 2(a)–2(c)).

**Figure 3:**
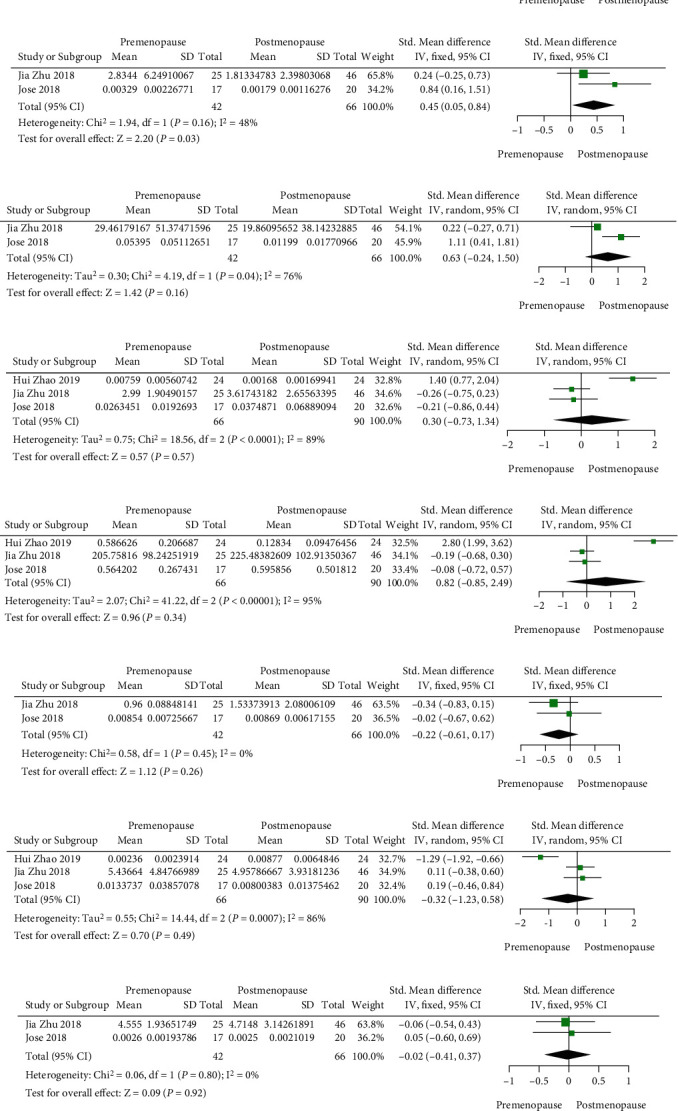
(a) *Odoribacter*; (b) *Bilophila*; (c) *Prevotella*; (d) *Parabacteroides*; (e) *Bacteroidetes*; (f) *Sutterella*; (g) *Roseburia*; (h) *Blautia.*

At the genus level, the abundance of *Odoribacter* (SMD = 0.83, 95%CI (0.43, 1.24), *P* < 0.0001) and *Bilophila* (SMD = 0.45, 95%CI (0.05, 0.84), *P* = 0.03) is higher in postmenopausal women. There are no differences in other bacteria, though *Prevotella* (MD = 0.63, 95%CI (−0.24, 1.50), *P* = 0.16), *Parabacteroides* (SMD = 0.30, 95%CI (−0.73, 1.34), *P* = 0.57), and *Bacteroidetes* (SMD = 0.82, 95%CI (−0.85, 2.49), *P* = 0.34) presented an upward trend, while *Sutterella* (SMD = −0.22, 95%CI (−0.61, 0.17), *P* = 0.26), *Roseburia* (SMD = −0.32, 95%CI (−1.23, 0.58), *P* = 0.49), and *Blautia* (SMD = −0.02, 95%CI (−0.41, 0.37), *P* = 0.92) showed a downward trend after menopause (Figures 3(a)–3(h)).

### 3.5. Genus Level Subgroup Analysis

**Figure 4:**
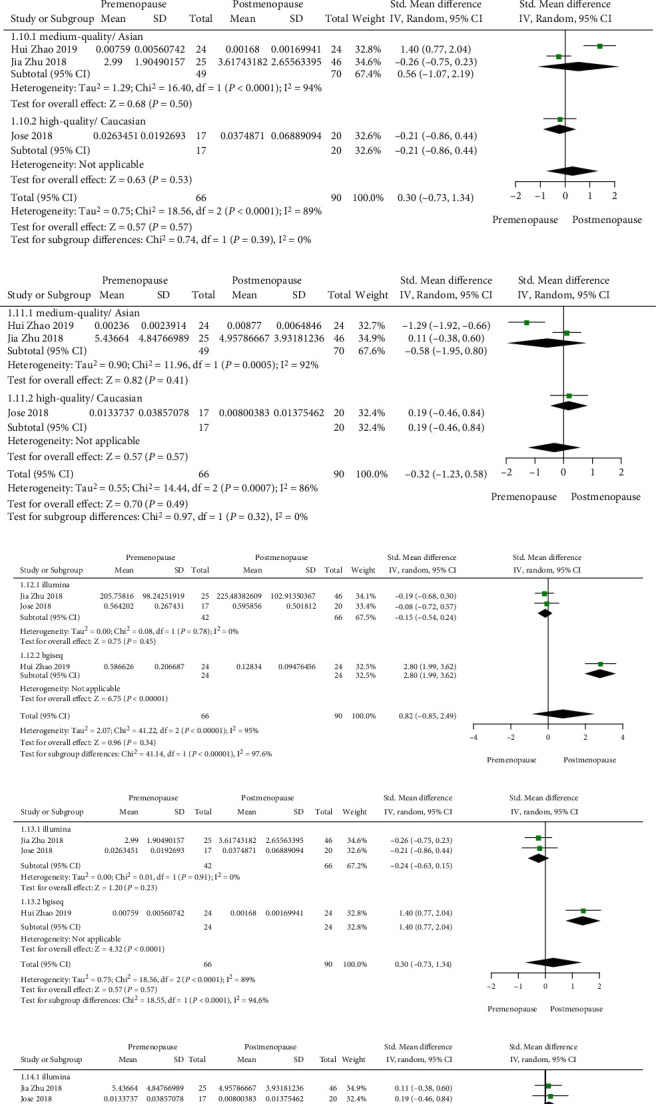
(a) *Bacteroides* (subgroup for race or NOS score); (b) *Parabacteroides* (subgroup for race or NOS score); (c) *Roseburia* (subgroup for race or NOS score); (d) *Bacteroides* (subgroup for method); (e) *Parabacteroides* (subgroup for method); (f) *Roseburia* (subgroup for method).

According to the quality of the article, the subgroup analysis of medium and high-quality studies was carried out, and the results showed that there was no difference in *Bacteroides, Parabacteroides*, and *Roseburia*. Similarly, there was no difference between Asian and European Americans after subgroup analysis. It shows that the research quality and race are not the sources of heterogeneity. According to the sequencing technology, it is divided into the Illumina group and the BGISEQ group. There are some differences between different sequencing technologies, which may be the source of heterogeneity (Figure 4).

### 3.6. Change in Intestinal Flora Diversity

**Figure 5:**
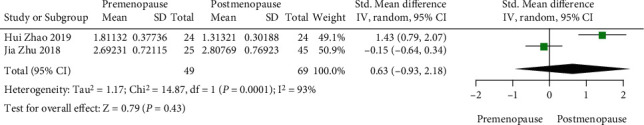
Shannon index.

The quantitative combination of the Shannon index of the two literatures showed that the species diversity increased after menopause, but the difference was not statistically significant (SMD = 0.63, 95%CI (−0.93, 2.18), *P* = 0.43) (Figure 5). Only one article reported the Chao1 index: premenopausal women (MD = 237.631, SD = 82.404) and postmenopausal women (MD = 205.052, SD = 136.062).

### 3.7. Relationship between Intestinal Flora and Estradiol

Two studies reported the relationship between intestinal flora and estradiol. We homogenized the unit into the Pearson correlation coefficient. Santos-Marcos et al. found that the serum estradiol level was positively correlated with the *Gammaproteobacteria* class (*R* = 0.575, *P* = 0.013) and an unknown genus from *Myxococcales* (*R* = 0.521, *P* = 0.039) and negatively correlated with *Prevotellaceae* (*R* = −0.523, *P* = 0.018). The estradiol level is positively correlated with *Shewanella putrefaciens* and *Erwinia amylovora*, but the correlation was weak, and the correlation coefficients were 0.394 (*P* = 0.025, *q* > 0.05) and 0.139 (*P* = 0.039, *q* > 0.05), respectively.

### 3.8. Metaregression

Metaregression was used to find potential sources of heterogeneity. When the quality of articles (high and medium quality), region (Asian and European), and the average age difference between groups (≤5 or >5) were used as preset factors, no source of heterogeneity was found (Table 3).

### 3.9. Sensitivity Analysis

**Figure 6:**
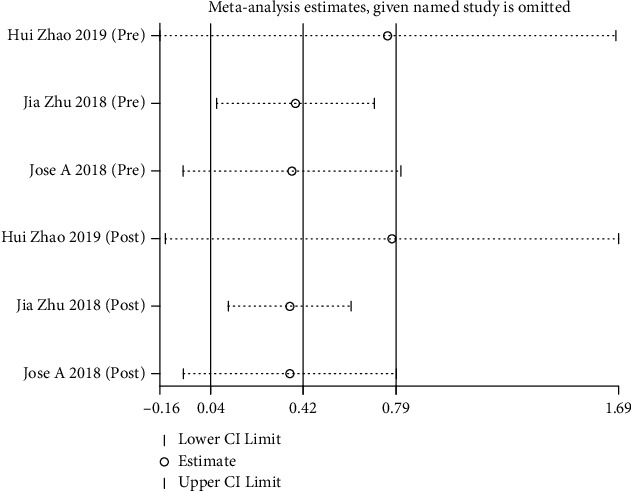
Sensitivity analysis.

Sensitivity analysis found that Zhao et al.'s study had a high degree of heterogeneity. Through comprehensive investigation, it is found that different sequencing technologies may lead to heterogeneity. After excluding the study, *I*^2^ decreased from 57% to 0%, and the results remained unchanged (SMD = 0.11, 95% CI (-0.39, 0.60)) (Figure 6).

### 3.10. Publication Bias

Only three articles were included in this study, and the funnel chart has a limited effect on the evaluation. Therefore, the funnel chart analysis was not carried out, and there may be some publication bias.

## 4. Discussion

There is no systematic review or meta-analysis of the changes in intestinal flora in postmenopausal women, and this is the first relevant meta-analysis. After a detailed search in seven databases, three literatures were included in this paper. Among the included case-control studies, two studies were rated as medium [5, 12] and one study as high quality by the NOS score.

The main results showed that at the phylum level, there were no differences in *Firmicutes*, *Bacteroidetes*, and *Proteobacteria* during menopause, while at the genus level, the relative abundance of *Odoribacter* and *Bilophila* in postmenopausal women was higher than that in premenopausal women. No differences were found in other bacteria. Regardless of that, *Sutterella*, *Roseburia*, and *Blautia* showed a downward trend after menopause, while *Prevotella*, *Parabacteroides*, and *Bacteroidetes* presented an upward trend. There was a positive correlation between estradiol and intestinal flora *Gammaproteobacteria*, especially *Shewanella putrefaciens* and *Erwinia amylovora*.

Hydrogen sulfide is produced by *Bilophila*. The physiological function of hydrogen sulfide is to relax ileal smooth muscle and increase the blood supply of gastrointestinal mucosa [12]. The increase of intestinal *Bilophila* in postmenopausal women leads to the increased production of hydrogen sulfide, which may lead to local inflammation and mucosal damage and then induce the increase of endotoxin in the serum and the inflammatory reaction of various tissues. The intracellular inflammatory response caused by inflammatory factors can lead to the obstruction of insulin signal transduction, resulting in insulin resistance. In bone, most inflammatory factors (such as TNF-*α*, IL-1, and IL-6) can promote the function of osteoclasts, thus breaking the balance between bone formation and absorption, resulting in osteopenia. In addition, inflammatory factors in peripheral blood can activate microglia of the central nervous system through the blood-brain barrier, cause inflammation of nerve cells, and exacerbate the aggregation and accumulation of nerve fiber tangles and *β*-amyloid protein, inducing Alzheimer's disease. Therefore, the increase of *Bilophila* in postmenopausal women may be related to osteoporosis, metabolic abnormalities, and Alzheimer's disease.

The increase of *Odoribacter* in postmenopausal women contributes to the increase of short-chain fatty acids (SCFAs), hydrogen, and hydrogen sulfide [13, 14]. Short-chain fatty acids can (1) increase fatty acid oxidation and energy metabolism, (2) participate in regulating the synthesis of serotonin and stabilizing neurons, and (3) increase circulating insulin-like growth factor-1 (IGF-1) which facilitates bone formation. Therefore, the increase of short-chain fatty acids caused by *Odoribacter* may ameliorate obesity, hyperlipidemia, depression, and osteoporosis in postmenopausal women. However, hydrogen sulfide produced by it can also cause an inflammatory reaction. Thus, *Odoribacter* may not only show a certain protective effect on postmenopausal women but also induce symptoms related to postmenopausal syndrome. The total effect remains unknown.

The first study on the differences in intestinal flora in postmenopausal women was conducted in Sweden in 2011. The bacteria were identified by multiplex PCR and partial 16s rDNA sequencing. The flora they tested was only Lactobacillus [15]. In the past decade, the development of gene sequencing technology and the establishment of databases have led to the rapid development of intestinal flora research. Most of the existing studies are intervention studies on postmenopausal women or ovariectomized female rats. In a few studies, there is no definite conclusion on the differences in the abundance of intestinal flora and the specific bacteria related to sex hormones. The reasons may be caused by races, inclusion criteria, detection methods, and so on.

The heterogeneity of intestinal flora related to the meta-analysis mainly comes from two aspects: one is the technical aspect, such as experimental design schemes, recruitment strategies, detection methods, and reference databases; the other is biology, such as the internal difference of community composition and the mobility of microorganisms to different environments. The source of heterogeneity in this paper may be sequencing techniques. Although studies have shown that the BGISEQ-500 platform is highly consistent with Illumina HiSeq2000 and Illumina HiSeq4000 platforms, data from different platforms should still be treated with caution [16]. Subgroup analysis elucidates that the heterogeneity between two studies using Illumina as a sequencing instrument is negligible, while it in BGISEQ is obvious, indicating that the use of a sequencing instrument may be one of the sources of heterogeneity.

At present, there is no systematic review of the changes in intestinal flora in menopause. This paper is a preliminary exploration of the intestinal flora differences in pre- and postmenopausal women. The lack of original articles, low quality, and large heterogeneity also bring us challenges in the meta-analysis. There are few studies that can be included for a few original studies. Therefore, we should be cautious about the results of this paper, and high-quality experiments are urgently needed.

Taken together, this meta-analysis systematically summarizes the changes in intestinal flora in pre- and postmenopausal women. The conclusion of our study may provide clues to the mechanism of menopausal syndrome and support basis for possible treatment methods such as probiotics and fecal microbiota transplantation.

## Figures and Tables

**Table 1:** Characteristics of included studies.

Study	Country	Years for menopause	Premenopause		Postmenopause		Outcome	NOS
			*n*	Mean age (y)	*n*	Mean age (y)		
Jose 2018 [4]	Spain	6.38 ± 1.14	17	46.12 ± 0.82	20	55.85 ± 0.66	①	7
Zhu 2018 [5]	China	8.19 ± 3.54	25	35.52 ± 6.02	46	56.89 ± 6.41	①②③	4
Zhao 2019 [12]	China	Natural menopause	24	52.55 ± 5.56	24	53.88 ± 3.81	②	6

*N*: number analyzed; ^①^abundance of bacteria; ^②^Shannon index; ^③^Chao1 index; NOS: Newcastle-Ottawa Scale case-control study score.

**Table 2:** Sample collection and treatment methods included in the study.

Study	Sample	Storage conditions	DNA extraction	Sequencing	Region for sequence	Database	Multiple comparisons
Jose 2018	Feces	NM	QIAamp DNA kit Stool Mini Kit (Qiagen)	Illumina MiSeq	V1-V2	Greengenes	Yes
Zhu 2018	Feces	-80°C	QIAamp DNA Stool	Illumina HiSeq	Metagenome	Virulence Factors of Pathogenic Bacteria database	Yes
Zhao 2019	Feces	-80°C	NM	BGISEQ-500	Metagenome	NM	No

NM: not mentioned.

**Table 3:** Metaregression.

	Bacteria	*N*	*I* ^2^ (%)	Adjusted *R*^2^ (%)	Coefficient	SE	*t*	*P*	95% CI
NOS score or race	Bacteroides (pre)	3	99.09	-300000	-101.47	177.48	-0.57	0.669	(-2356.56, 2153.62)
	Bacteroides (post)	3	99.55	-0.52	-111.70	194.87	-0.57	0.669	(-2587.73, 2364.33)
	Parabacteroides (pre)	3	98.37	-22000	-1.45	2.57	-0.56	0.673	(-34.09, 31.19)
	Parabacteroides (post)	3	98.83	-0.5527	-1.75	3.12	-0.56	0.674	(-41.38, 37.88)
	Roseburia (pre)	3	96.82	-720000	-2.62	4.66	-0.56	0.674	(-61.77, 56.53)
	Roseburia (post)	3	98.63	0.0	-2.44	4.27	-0.57	0.669	(-56.65, 51.77)

Age difference	Bacteroides (pre)	3	99.08	-300000	102.03	177.16	0.58	0.667	(-2148.96, 2353.02)
	Bacteroides (post)	3	99.54	-0.51	112.40	194.46	0.58	0.666	(-2358.48, 2583.28)
	Parabacteroides (pre)	3	98.35	-22000	1.48	2.55	0.58	0.666	(-30.95, 33.91)
	Parabacteroides (post)	3	98.80	-0.52	1.80	3.09	0.58	0.663	(-37.43, 41.04)
	Roseburia (pre)	3	96.80	-720000	2.64	4.65	0.57	0.671	(-56.39, 61.66)
	Roseburia (post)	3	98.53	0.0	2.44	4.27	0.57	0.669	(-51.78, 56.66)

*N*: number of study.

## Data Availability

All data was provided in the manuscript.
